# Lifestyle, chronic diseases and self-rated health among Malaysian adults: results from the 2011 National Health and Morbidity Survey (NHMS)

**DOI:** 10.1186/s12889-015-2080-z

**Published:** 2015-08-06

**Authors:** Ying Ying Chan, Chien Huey Teh, Kuang Kuay Lim, Kuang Hock Lim, Pei Sien Yeo, Chee Cheong Kee, Mohd Azahadi Omar, Noor Ani Ahmad

**Affiliations:** Institute for Public Health, National Institutes of Health, Ministry of Health Malaysia, Kuala Lumpur, Malaysia; Institute for Medical Research, National Institutes of Health, Ministry of Health Malaysia, Kuala Lumpur, Malaysia

**Keywords:** Self-rated health, lifestyle, chronic diseases, National Health and Morbidity Survey, Malaysia

## Abstract

**Background:**

Self-rated health (SRH) has been demonstrated as a valid and appropriate predictor of incident mortality and chronic morbidity. Associations between lifestyle, chronic diseases, and SRH have been reported by various population studies but few have included data from developing countries. The aim of this study was to determine the prevalence of poor SRH in Malaysia and its association with lifestyle factors and chronic diseases among Malaysian adults.

**Methods:**

This study was based on 18,184 adults aged 18 and above who participated in the 2011 National Health and Morbidity Survey (NHMS). The NHMS was a cross-sectional survey (two-stage stratified sample) designed to collect health information on a nationally representative sample of the Malaysian adult population. Data were obtained via face-to-face interviews using validated questionnaires. Two categories were used to measure SRH: “good” (very good and good) and “poor” (moderate, not good and very bad). The association of lifestyle factors and chronic diseases with poor SRH was examined using univariate and multivariate logistic regression.

**Results:**

Approximately one-fifth of the Malaysian adult population (20.1 %) rated their health as poor (men: 18.4 % and women: 21.7 %). Prevalence increases with age from 16.2 % (aged 18–29) to 32.0 % (aged ≥60). In the multivariate logistic regression analysis, lifestyle factors associated with poor SRH included: underweight (OR = 1.29; 95 % CI: 1.05–1.57), physical inactivity (OR = 1.25; 95 % CI: 1.11–1.39), former smoker (OR = 1.38; 95 % CI: 1.12–1.70), former drinker (OR = 1.27; 95 % CI: 1.01–1.62), and current drinker (OR = 1.35; 95 % CI: 1.08–1.68). Chronic diseases associated with poor SRH included: asthma (OR = 1.66; 95 % CI: 1.36–2.03), arthritis (OR = 1.87; 95 % CI: 1.52–2.29), hypertension (OR = 1.39; 95 % CI: 1.18–1.64), hypercholesterolemia (OR = 1.43; 95 % CI: 1.18–1.74), and heart disease (OR = 1.85; 95 % CI: 1.43–2.39).

**Conclusions:**

This study indicates that several unhealthy lifestyle behaviours and chronic diseases are significantly associated with poor SRH among Malaysian adults. Effective public health strategies are needed to promote healthy lifestyles, and disease prevention interventions should be enhanced at the community level to improve overall health.

**Electronic supplementary material:**

The online version of this article (doi:10.1186/s12889-015-2080-z) contains supplementary material, which is available to authorized users.

## Background

A variety of lifestyle factors and health-related behaviours such as smoking, alcohol consumption, physical activity, and dietary habits can affect a person’s health. An unhealthy lifestyle often results in a higher risk of chronic diseases such as heart disease, hypertension, hypercholesterolemia, and diabetes [1, 2]. Previous empirical studies have found that lifestyle factors and chronic diseases are strongly associated with self-rated health (SRH) [3–5]. Several socio-environmental determinants such as infrastructure, housing conditions, political situation, access to health care services, availability of clean water, and affordability of healthy foods are also found to be closely related to SRH [6–8]. SRH is an established predictor of morbidity and mortality and has been widely used to measure health inequalities in public health research [9, 10]. It has been recognised as a reliable and valid health indicator that is based on a simple question in which respondents are asked to rate their current general health status on a four- or five-point scale ranging from “very good (excellent)” to “very poor” [9]. The SRH is frequently used in population health surveys because the data are easily collected; SRH has been extensively studied in Western populations [11].

Understanding the correlates of SRH can help public health professionals prioritise health-promotion and disease-prevention interventions. To date, studies that have investigated the determinants of SRH have focused primarily on Western countries, although we have found a amall number of studies that examine the topic from the perspective of developing countries in the Asian regions. Previous studies from Singapore [12], Pakistan [13], and Japan [14] have found that physical activity, tobacco use, and BMI have shown a significant association with self-reported fair or poor health. Another study from Thailand reported that chronic diseases, functional status, and psychosocial symptoms were the strongest determinants of poor SRH among Thai elderly [15]. The increasing research interest in SRH in Asian developing countries warrants this study of SRH in Malaysia, not only to provide data on SRH comparable to that of neighbouring countries such as Thailand and Singapore, but also to provide evidence-based results for developing appropriate public health policies and programmes that improve the overall health of the population.

In Malaysia, few studies have examined the topic of SRH. A recent study by Cheah [16] reported that socio-demographic characteristics, lifestyle factors, and chronic diseases have a substantial impact on SRH. However, the data were from only one state (Penang) and may not be representative of the country as a whole. Other studies focus only on exploring the socio-demographic determinants of SRH among the elderly [17, 18]. As such, the information gathered from these studies is not sufficient for policy implementation at the national level. Hence, the present study aims to fill the gaps and improve the existing knowledge on determinants of SRH among the general population in Malaysia. Using data from the 2011 National Health and Morbidity Survey (NHMS), we determined the prevalence of poor SRH and its possible determinants—with an emphasis on relationships between lifestyle factors, chronic diseases, and SRH—in a large sample of the Malaysian adult population.

## Methods

### Study design and data collection

The NHMS 2011 was a nationwide, cross-sectional, population-based survey that used a two-stage stratified sampling design to select a nationally representative sample. The Malaysian states constituted the primary strata, with further stratification by urban–rural residence. The primary sampling units were enumeration blocks (EBs) provided by the Malaysian Department of Statistics from the 2010 census. Living quarters (LQs) represent the secondary sampling units. A total of 794 EBs (484 urban and 310 rural) were systematically selected from all the Malaysian EBs (about 75,000) via a probability-proportional-to-size sampling technique. Subsequently, 12 LQs were randomly selected from the approximately 80 to 120 LQs in each EB. Finally, all households and eligible household members in the selected LQs were included in the sample. The survey methodology is described in greater detail by Fadhli et al. [19].

Data collection for the NHMS 2011 was carried out from April to July 2011. Information on SRH, socio-demographic characteristics, lifestyle, and self-reported medically-diagnosed chronic diseases was obtained by trained interviewers via face-to-face interviews. The interviewers were extensively trained and conducted mock interviews prior to actual data collection. The interviewing process was closely monitored by field supervisors, who were health care personnel. A total of 18,231 eligible adults aged 18 years and older, living in the sampled households, were interviewed. Of these, 18,184 responded to the SRH questionnaire section of the NHMS 2011, giving a response rate of 99.7 %. To ensure a high response rate, unoccupied households were revisited up to three times. All of the eligible respondents provided written consent to be interviewed for the survey. The study protocol was approved by the Malaysian Medical and Research Ethics Committee (MREC), Ministry of Health Malaysia (NMRR-10-757-6837).

### Measures

#### Dependent variable

Self-rated health was measured based on the question: “In general, how would you rate your health today?” There were five response categories: “very good”, “good”, “moderate”, “not good” and “very bad”. For purposes of analysis, the response categories were combined into two groups: “Good”, which included “very good” and “good”, and “Poor” which included “moderate”, “not good” and “very bad”. “Good” SRH served as the reference category in the logistic regression analyses.

#### Independent variables

Socio-demographic variables included sex, age, ethnicity, residential area (urban/rural), marital status (single, married, widow/widower/divorcee), educational level (no formal education, primary, secondary, tertiary), current work status (working/not working) and monthly household income. Age was categorised into five groups: 18–29 years, 30–39 years, 40–49 years, 50–59 years, 60 years and above. Ethnicity was categorised into five groups: Malays, Chinese, Indians, other Bumiputeras (including indigenous Orang Asli in Peninsular Malaysia and the indigenous groups in Sabah and Sarawak such as Kadazan, Murut, Bajau, Melanau, Iban and Bidayuh), and “Others” (including small minority groups that settled in Malaysia such as Sikh, Serani, Portuguese, Indonesian, and Bangladeshi). Monthly household income was grouped into four levels: less than MYR1000, MYR1000–MYR1999, MYR2000–MYR2999, MYR3000 and above [1 Malaysian Ringgit (MYR) ≈ 0.29 US Dollar (USD)].

Lifestyle variables included body mass index (BMI), consumption of fruits and vegetables, physical activity, smoking, and alcohol consumption. Respondents’ weight and height were measured using the Tanita 318 digital weighing scale (Tanita Corp., Tokyo, Japan) and the Seca 206 Bodymeter measuring tape (seca GmbH & Co. KG, Hamburg, Germany), to the nearest 0.1 kg and 0.1 cm, respectively. BMI was calculated as weight divided by height in meters squared (kg/m^2^). According to the World Health Organization (WHO 2000) guidelines [20], BMI was categorised according to four levels of nutritional status for both men and women: underweight (<18.5), normal (18.5–24.9), overweight (25.0–29.9) and obese (≥30).

Consumption of fruits and vegetables was measured based on four questions: i) “In a typical week, how many days do you eat fruits?” ii) “Usually on the day you eat fruits, how many servings of fruits did you eat in a day?” iii) “In a typical week, how many days do you eat vegetables”, and iv) “Usually on the day you eat vegetables, how many servings of vegetables did you eat in a day?” Photographs of foods were used to help respondents recall the serving sizes of the fruits and vegetables they had consumed. The photographs showed single servings of commonly consumed fruits and vegetables such as the following: one medium-sized apple, one medium-sized banana, one slice of papaya, one slice of watermelon, eight medium-sized grapes, one cup of chopped raw leafy green vegetables or *ulam* (Malaysian salad herbs), and a half cup of cooked chopped eggplant or tomato or carrot or leafy green vegetables. According to the WHO STEPS criteria [21], responses to these four questions were used to obtain an assessment of total average consumption of fruits and vegetables per day, without differentiating between the two categories. Based on the WHO recommendations [22], consumption of fruits and vegetables was divided into two groups: adequate consumption of fruits and vegetables (≥5 servings per day), and inadequate consumption of fruits and vegetables (<5 servings per day).

The level of physical activity was determined based on the short form of the International Physical Activity Questionnaire (IPAQ), the reliability and validity of which have been established [23]. The original English version of the questionnaire was translated into Bahasa Malaysia, then back-translated into English for verification. The Malay version of IPAQ was pilot tested on 32 adults for concurrent validity (correlation with the original English version) by comparing the correlation of total physical activity from the Malay version with the one obtained from the original English version. An interval of one hour was allowed between administration of the two questionnaires and a good concurrent validity with Spearman correlation coefficient (ρ) of 0.995 for total physical activity was obtained. The IPAQ short form was designed to estimate the overall physical activity level by assessing three specific types of physical activity (walking, moderate-intensity activities and vigorous-intensity activities) undertaken across a comprehensive set of domains (work-related, transportation, leisure time, and domestic/gardening). The level of physical activity is calculated in terms of metabolic energy expended (MET minutes, or METs), based on the IPAQ analysis protocol [24]. The total physical activity score is the sum of all METs per week from walking to moderate-intensity activities to vigorous-intensity activities. Survey participants who performed any combination of walking, moderate-intensity activities or vigorous-intensity activities in the past 7 days, with a total physical activity score of at least 600 METs per week were categorised as “active”. Those who achieved less than 600 METs per week were classified as “inactive”.

Smoking status was divided into three categories: “never smoker” (never smoked in lifetime), “former smoker” (smoked daily or occasionally in the past but not a smoker at the time of the survey), and “current smoker” (smoked any tobacco products either daily or occasionally at the time of the survey). Alcohol consumption status was divided into three categories: “non-drinker” (no alcohol consumption for past 12 months, includes never drinkers), “former drinker” (consumed alcohol in the past but had stopped drinking for the past 12 months) and “current drinker” (consumed alcohol in the past 12 months).

For chronic disease status, presence or absence of chronic diseases was based on self-reported information on chronic conditions diagnosed by a doctor or health care professional. Respondents were asked: “Have you ever been told by a doctor or Medical Assistant that you have __________?” followed by a list of chronic diseases included asthma, arthritis, diabetes mellitus, hypertension, hypercholesterolemia, and heart disease, to which the answer is “yes” or “no”.

### Statistical analyses

Frequency distributions of socio-demographic, lifestyle, and chronic disease variables were analysed by self-rated health status. Descriptive statistics were used to calculate the overall prevalence of poor SRH among Malaysian adults and the prevalence of poor SRH by socio-demographic, lifestyle and chronic disease variables. Associations of the measured factors with poor SRH were tested using chi-square tests. Univariate and multivariate logistic regression analyses were used to investigate the association between poor SRH, socio-demographic, lifestyle, and chronic disease variables, with “Good” SRH as the reference category. The results are presented as crude and adjusted odds ratios (ORs) with 95 % confidence intervals (CIs). Variables that were associated with poor SRH at *p* = <0.25 in the univariate analysis were retained in the multivariate logistic regression model [25]. Those variables (i.e., “Others” ethnic group, overweight BMI group, and current drinkers) that by themselves were not significantly related to poor SRH in the univariate analysis, but were considered to make an important contribution in the presence of other variables, were also included in the final model. The multivariate logistic regression model was adjusted for all potential confounding factors and *p* = <0.05 was considered to be statistically significant. The statistical analyses were performed using SPSS for Windows (version 20.0) statistical software (IBM Corp., Armonk, NY, USA). All analyses were conducted using a complex sampling design to ensure that sample weights and study design were taken into consideration.

## Results

Table 1 shows the distribution of socio-demographic characteristics, lifestyle factors, and chronic disease variables across the two categories of SRH (“good” and “poor”) in the study sample (*n* = 18,184). The sample included more women (53.4 %) than men (46.6 %). Among the 18,184 respondents, the distribution of self-rated health was as follow: “very good” 3,468 (19.4 %); “good” 11,031 (60.4 %); “moderate” 3,349 (18.2 %); “not good” 316 (1.8 %); and “very bad” 20 (0.1 %) (see Additional file 1: Table S1). We combined the five categories into two, “good” (“very good” and “good”) and “poor” (“moderate”, “not good,” and “very bad”) SRH, with 79.9 % (*n* = 14,499) of participants reporting good SRH, and 20.1 % (*n* = 3685) reporting poor SRH. A higher proportion of women (57.5 %) than men (42.5 %) reported poor SRH. Similarly, a higher proportion of older adults (aged 60 years and above) reported poor SRH than younger adults (Table 1).

**Table 1:** Distribution of socio-demographic characteristics, lifestyle factors, and self-reported medically-diagnosed chronic diseases, by self-rated health status, NHMS 2011

Variables	Total (n=18184)	Self-rated health status	
		Good (n=14499)	Poor (n=3685)
	n (%)	n (%)	n (%)
Sex			
Men	8588 (46.6)	7023 (47.7 %)	1565 (42.5 %)
Women	9596 (53.4)	7476 (52.3 %)	2120 (57.5 %)
Age group (years)^a^			
18–29	4599 (26.4)	3871 (27.9)	728 (20.6)
30–39	3452 (20.2)	2844 (20.8)	608 (17.7)
40–49	3441 (20.5)	2735 (20.5)	706 (20.0)
50–59	2981 (17.4)	2332 (17.4)	649 (17.5)
≥60	2687 (15.6)	1822 (13.4)	865 (24.2)
Ethnicity			
Malays	10,309 (54.6)	8143 (54.0)	2166 (57.2)
Chinese	3546 (20.5)	2976 (21.4)	570 (16.8)
Indians	1446 (8.4)	1159 (8.5)	287 (7.8)
Other Bumiputeras	1802 (11.6)	1313 (11.1)	489 (13.6)
Others	1081 (4.9)	908 (5.0)	173 (4.6)
Residential area			
Urban	10,116 (58.1)	8238 (59.4)	1878 (53.1)
Rural	8068 (41.9)	6261 (40.6)	1807 (46.9)
Marital status^a^			
Single	4905 (26.7)	4166 (28.4)	739 (19.9)
Married	11,851 (65.5)	9330 (64.6)	2521 (68.9)
Widow/widower/divorcee	1419 (7.8)	997 (7.0)	422 (11.1)
Education level^a^			
No formal education	1442 (8.0)	1007 (7.0)	435 (12.0)
Primary	4296 (23.8)	3256 (22.7)	1040 (28.1)
Secondary	8703 (49.0)	7106 (50.3)	1597 (43.8)
Tertiary	3395 (19.2)	2838 (20.0)	557 (16.1)
Currently working^a^			
Yes	10,689 (59.3)	8767 (60.6)	1922 (54.3)
No	7484 (40.7)	5724 (39.4)	1760 (45.7)
Monthly household income *(1 MYR ≈ 0.29 USD)*			
<RM1000	3458 (17.1)	2712 (16.9)	746 (18.0)
RM1000-RM1999	3669 (18.2)	2932 (18.0)	737 (18.8)
RM2000-RM2999	3084 (16.8)	2463 (16.9)	621 (16.6)
≥RM3000	7973 (47.9)	6392 (48.1)	1581 (46.6)
BMI status (kg/m^2^)^a^			
Underweight (<18.5)	1211 (7.8)	954 (7.5)	257 (8.7)
Normal (18.5–24.9)	7189 (45.5)	5868 (46.1)	1321 (42.9)
Overweight (25.0–29.9)	4842 (30.6)	3874 (30.7)	968 (30.0)
Obese (≥30)	2593 (16.2)	1971 (15.7)	622 (18.3)
Consumption of fruits and vegetables^a^			
<5 servings/day	16,844 (92.8)	13,402 (92.6)	3442 (93.4)
≥5 servings/day	1314 (7.2)	1075 (7.4)	239 (6.6)
Physical activity status^a^			
Active	11,570 (64.0)	9472 (65.9)	2098 (56.7)
Inactive	6476 (36.0)	4914 (34.1)	1562 (43.4)
Smoking status^a^			
Never smoker	12,769 (70.8)	10,199 (70.9)	2570 (70.2)
Former smoker	1215 (7.1)	864 (6.4)	351 (9.8)
Current smoker	4069 (22.1)	3334 (22.7)	735 (20.0)
Drinking status^a^			
Non-drinker	15,482 (84.5)	12,374 (84.6)	3108 (84.0)
Former drinker	959 (5.6)	723 (5.4)	236 (6.2)
Current drinker	1642 (9.9)	1325 (10.0)	317 (9.9)
Presence of chronic disease^b^			
Asthma	1142 (6.3)	781 (5.5)	361 (9.9)
Arthritis	985 (5.5)	577 (4.2)	408 (10.3)
Diabetes mellitus	1546 (9.3)	1082 (8.5)	464 (12.3)
Hypertension	2679 (15.6)	1820 (13.6)	859 (23.6)
Hypercholesterolemia	1578 (9.4)	1058 (8.0)	520 (14.5)
Heart disease	612 (3.1)	356 (2.3)	256 (6.4)

^a^Differences in ‘N’ values are due to missing data on variables.

^b^‘N’ values and percentages shown refer only to respondents who reported the presence of chronic disease.

Table 2 shows the prevalence of poor SRH by socio-demographic characteristics, lifestyle factors, and chronic disease variables. Overall, the prevalence of poor SRH was higher among women (21.7 %) than men (18.4 %). The prevalence of poor SRH increased with age and was highest in the oldest age group ≥60 years (32.0 %). A higher prevalence of poor SRH was also found among Other Bumiputeras, rural residents, widows/widowers/divorcees, individuals with no formal education, and those who were currently not working. Prevalence of poor SRH was significantly related to sex, age, ethnicity, residential area, marital status, education level, current work status, BMI status, physical activity status, smoking status, and chronic disease status (*p* = <0.001). Regarding lifestyle variables, individuals who were underweight, physically inactive, and former smokers had higher prevalence of poor SRH. Survey participants who had chronic diseases such as asthma, arthritis, diabetes mellitus, hypertension, hypercholesterolemia, and heart disease were found to have a higher prevalence of poor SRH, compared with their counterparts who did not have these diseases.

**Table 2:** Prevalence of poor self-rated health by socio-demographic characteristics, lifestyle factors, and chronic disease status among Malaysian adults aged 18 years and above, NHMS 2011

Variables	Prevalence (%)	95 % CI	*p-*value
SOCIO-DEMOGRAPHIC CHARACTERISTICS			
Sex			<0.001
Men	18.4	17.2–19.6	
Women	21.7	20.5–22.9	
Total	20.1	19.2–21.1	
Age group (years)			<0.001
18–29	16.2	14.7–17.8	
30–39	18.1	16.3–20.0	
40–49	20.2	18.5–22.1	
50–59	20.7	18.8–22.8	
≥60	32.0	29.6–34.6	
Ethnicity			<0.001
Malays	21.1	20.0–22.2	
Chinese	16.6	14.9–18.3	
Indians	18.7	15.8–22.1	
Other Bumiputeras	23.6	20.3–27.2	
Others	18.8	15.2–23.0	
Residential area			<0.001
Urban	18.4	17.2–19.6	
Rural	22.6	21.1–24.0	
Marital status			<0.001
Single	15.0	13.6–16.5	
Married	21.2	20.1-22.3	
Widow/widower/divorcee	28.6	25.6–31.7	
Educational level			<0.001
No formal education	30.5	27.2–33.9	
Primary education	23.9	22.1–25.7	
Secondary education	18.1	16.9–19.2	
Tertiary education	16.9	15.3-18.7	
Current work status			<0.001
Working	18.4	17.3–19.7	
Non-working	22.6	21.3–24.0	
Monthly household income *(1 MYR ≈ 0.29 USD)*			0.530
<MYR1000	21.1	19.2–23.2	
MYR1000-MYR1999	20.8	19.0–22.6	
MYR2000-MYR2999	19.9	18.0-21.9	
≥MYR3000	19.6	18.3–21.0	
LIFESTYLE FACTORS			
BMI status (kg/m^2^)			<0.001
Underweight (<18.5)	22.5	19.4–25.8	
Normal (18.5-24.9)	18.8	17.6–20.1	
Overweight (25.0–29.9)	19.6	18.0-21.2	
Obese (≥30)	22.4	20.4–24.6	
Consumption of fruits and vegetables			0.235
<5 servings/day	20.3	19.3–21.3	
≥5 servings/day	18.4	15.6–21.5	
Physical activity status			<0.001
Active	17.8	16.7–18.9	
Inactive	24.3	22.8–25.8	
Smoking status			<0.001
Never smoker	20.0	18.9–21.0	
Former smoker	27.9	24.8–31.2	
Current smoker	18.1	16.5–19.8	
Drinking status			0.474
Non-drinker	20.0	19.0–21.0	
Former drinker	22.3	18.8–26.2	
Current drinker	19.9	17.2–22.9	
CHRONIC DISEASE STATUS			
Asthma			<0.001
Yes	31.3	27.9–34.8	
No	19.4	18.4–20.3	
Arthritis			<0.001
Yes	38.0	34.0–42.2	
No	19.1	18.2–20.0	
Diabetes mellitus			<0.001
Yes	27.4	24.4–30.5	
No	19.9	18.9–21.0	
Hypertension			<0.001
Yes	31.2	28.9–33.5	
No	19.5	17.7–19.7	
Hypercholesterolemia			<0.001
Yes	32.0	29.0–35.3	
No	19.5	18.5-20.5	
Heart disease			<0.001
Yes	41.5	36.5–46.6	
No	19.4	18.5-20.4	

Table 3 shows the results of univariate and multivariate logistic regression analyses for poor SRH by socio-demographic characteristics, lifestyle factors, and chronic disease variables. In the univariate analysis, all variables were associated with poor SRH at *p* = <0.25 except for “Others” ethnic group, monthly household income, overweight BMI group, and current drinkers. In the final multivariate logistic regression model, we included all variables that were statistically significant in univariate analysis (*p* = <0.25) except for the “monthly household income” variable. After adjusting for all selected factors in the multivariate analysis, the odds of poor SRH were significantly higher for women compared with men, among older people (aged ≥60 years) compared with younger people (aged 18–29 years), and among people living in rural areas compared with urban areas. Malays, other Bumiputeras and “Others” ethnic groups had higher odds of poor SRH compared with the Chinese. Respondents who were underweight (*p* = <0.05), physically inactive (*p* = <0.001), former smokers (*p* = <0.01), current drinkers (*p* = <0.01), and former drinkers (*p* = <0.05) reported significantly higher odds of poor SRH compared with their respective counterparts. Self-reported medically-diagnosed chronic diseases that were significantly associated with poor SRH were asthma, arthritis, hypertension, hypercholesterolemia, and heart disease (*p* = <0.001 for each). Individuals with diabetes mellitus were not significantly associated with poor SRH in our study.

**Table 3:** Univariate and multivariate logistic regression analyses for poor self-rated health, Malaysian adults aged 18 years and older, by socio-demographic characteristics, lifestyle factors, and chronic disease status

Variables	Crude OR (95 % CI)	*p*-value	Adjusted OR^a^ (95 % CI)	*p*-value
SOCIO-DEMOGRAPHIC CHARACTERISTICS				
Sex				
Men	1.00		1.00	
Women	1.23 (1.12–1.35)	<0.001	1.32 (1.15–1.52)	<0.001
Age group (years)				
18–29	1.00		1.00	
30–39	1.15 (0.98–1.35)	0.098	1.08 (0.89–1.32)	0.436
40–49	1.32 (1.13–1.54)	0.001	1.16 (0.95–1.43)	0.154
50–59	1.36 (1.16–1.58)	<0.001	1.05 (0.85–1.30)	0.653
≥60	2.44 (2.09–2.86)	<0.001	1.51 (1.17–1.93)	0.001
Ethnicity				
Malays	1.35 (1.17–1.54)	<0.001	1.52 (1.27–1.81)	<0.001
Chinese	1.00		1.00	
Indians	1.16 (0.91–1.48)	0.226	1.20 (0.92–1.57)	0.187
Other Bumiputeras	1.55 (1.25–1.93)	<0.001	1.62 (1.28–2.07)	<0.001
Others	1.17 (0.88–1.54)	0.280	1.46 (1.07–2.00)	0.018
Residential area				
Urban	1.00		1.00	
Rural	1.29 (1.15–1.45)	<0.001	1.15 (1.01–1.31)	0.032
Marital status				
Single	1.00		1.00	
Married	1.52 (1.35–1.71)	<0.001	1.09 (0.90–1.31)	0.413
Widow/widower/divorcee	2.26 (1.88–2.72)	<0.001	1.00 (0.76–1.32)	0.992
Educational level				
No formal education	2.15 (1.77–2.62)	<0.001	1.07 (0.81–1.40)	0.647
Primary education	1.54 (1.33–1.79)	<0.001	1.13 (0.93–1.36)	0.220
Secondary education	1.08 (0.95–1.24)	0.243	1.08 (0.93–1.26)	0.296
Tertiary education	1.00		1.00	
Current work status				
Working	1.00		1.00	
Not working	1.30 (1.17–1.43)	<0.001	0.94 (0.82–1.07)	0.337
Monthly household income *(1 MYR ≈ 0.29 USD)*				
<MYR1000	1.10 (0.95–1.26)	0.251	-	-
MYR1000-MYR1999	1.07 (0.94–1.23)	0.305	-	-
MYR2000-MYR2999	1.02 (0.88–1.18)	0.822	-	-
≥MYR3000	1.00		-	-
LIFESTYLE FACTORS				
BMI status (kg/m^2^)				
Underweight (<18.5)	1.25 (1.03–1.51)	0.022	1.29 (1.05–1.57)	0.014
Normal (18.5–24.9)	1.00		1.00	
Overweight (25.0-29.9)	1.05 (0.93–1.19)	0.417	0.94 (0.83–1.07)	0.356
Obese (≥30)	1.25 (1.09–1.43)	0.002	1.06 (0.91–1.23)	0.470
Consumption of fruits and vegetables				
<5 servings/day	1.13 (0.92–1.38)	0.235	1.10 (0.88–1.37)	0.412
≥5 servings/day	1.00		1.00	
Physical activity status				
Active	1.00		1.00	
Inactive	1.48 (1.34–1.63)	<0.001	1.25 (1.11–1.39)	<0.001
Smoking status				
Never smoker	1.00		1.00	
Former smoker	1.55 (1.31–1.84)	<0.001	1.38 (1.12–1.70)	0.003
Current smoker	0.89 (0.79–1.00)	0.047	1.08 (0.91–1.29)	0.356
Drinking status				
Non-drinker	1.00		1.00	
Former drinker	1.15 (0.92–1.43)	0.218	1.27 (1.01–1.62)	0.049
Current drinker	1.00 (0.83–1.20)	0.975	1.35 (1.08–1.68)	0.008
CHRONIC DISEASE STATUS				
Asthma				
Yes	1.89 (1.60–2.25)	<0.001	1.66 (1.36–2.03)	<0.001
No	1.00		1.00	
Arthritis				
Yes	2.60 (2.19–3.09)	<0.001	1.87 (1.52–2.29)	<0.001
No	1.00		1.00	
Diabetes mellitus				
Yes	1.51 (1.29–1.78)	<0.001	0.89 (0.72–1.09)	0.261
No	1.00		1.00	
Hypertension				
Yes	1.97 (1.77–2.20)	<0.001	1.39 (1.18–1.64)	<0.001
No	1.00		1.00	
Hypercholesterolemia				
Yes	1.95 (1.69–2.26)	<0.001	1.43 (1.18–1.74)	<0.001
No	1.00		1.00	
Heart disease				
Yes	2.94 (2.38–3.63)	<0.001	1.85 (1.43–2.39)	<0.001
No	1.00		1.00	

^a^Odds ratios (ORs) adjusted for all other variables shown in the table.

## Discussion

The prevalence of poor SRH in Malaysia (20.1 %) is similar to that reported in neighbouring Singapore (23.2 %) [12], which may be related to similarity of both ethnic composition and socio-economic characteristics. However, the level of poor SRH is much higher in Malaysia than in Japan (9.8 %) [26], Canada (11.9 %) [27] or the United States (16.4 %) [28]. Conversely, poor SRH is much lower in Malaysia than in Pakistan (65.1 %) [13]. Such comparisons across countries should be viewed with caution because of differences in the characteristics of the study populations (e.g., age range, gender, ethnic groups) and differences in the methodology used (e.g., type of rating scale, method used to elicit responses, and wording of questions) [29]. Additionally, differences in health status can be linked to different geographical, cultural, socio-economic, and socio-political factors, as well as other factors that affect perception of one’s self-rated health [30, 31].

The present study found a significant association between specific lifestyle factors and SRH. Individuals who are underweight, physically inactive, former smokers, and current drinkers were significantly associated with poor SRH. Previous studies have reported that obesity and underweight are significantly associated with poor SRH [13, 32, 33]; however, our study found that only underweight was significantly associated with poor SRH. This finding was in line with the result of a study in Singapore [34] that found no significant association between obesity and poor SRH. Conflicting findings have emerged regarding the association between BMI and SRH across different studies and populations. Two studies in Greece showed inconsistent findings between urban and rural populations, with obesity associated with poor SRH in urban populations [35] but no significant association between BMI and SRH in rural populations [36]. Another Greek study reported that obesity is associated with good SRH in centenarians [37]. The inconsistent association between BMI and SRH is partially related to differences in perceptions about the body and health in the study populations [38]. In our study, a possible explanation for the association between underweight and poor SRH among Malaysians is that people may be influenced by traditional ideas that being “heavy” (overweight/obesity) is “healthy,” while being underweight is not healthy [39].

Previous studies have consistently shown that physical activity and exercise have positive effects on SRH [40–43]. Our results generally support this finding by showing that individuals who were physically inactive were more likely to report poor SRH. It is consistent with previous findings from Malaysia reported by Cheah [16,] and also findings from Singapore reported by Ramkumar et al. [34]. The latter studied 409 Singaporean adults aged 40 years and above and found that lack of exercise was associated with poor SRH. Inconsistent findings were reported in a study conducted in Poland that found no positive association between physical activity and SRH [44]. The contradictory findings may be related to the Poland study [44] measuring physical activity only in terms of leisure time while our study measured *total physical activity* including different forms of physical activity (e.g., work-related, transportation, leisure-time, and domestic/gardening). A recent study of physical activity using the same national survey data (NHMS 2011) reported that about two in five Malaysian adults were physically inactive, particularly women and the elderly [45]. In the current study, these two groups were more likely to report poor SRH.

In contrast to findings from the 2007 Behavioural Risk Factor Surveillance System (BRFSS) survey conducted in the United States [46] and the Wellbeing, Eating and Exercise for a Long Life (WELL) study in Australia [47], our study did not show any relationship between consumption of fruits and vegetables and SRH. However, it should be noted that the survey methods, the questions used to measure consumption of fruits and vegetables, and the response options in the two studies were different from those used in our study. It is also possible that the measurement of servings of fruits and vegetables consumed per day was inaccurate because of self-reporting, which is subject to recall bias. However, our results were in line with studies by Darviri et al. [35] and Kwasniewska et al. [44], which reported no significant association between consumption of fruits and vegetables and SRH. The association between consumption of fruits and vegetables and SRH would benefit from further investigation using a more detailed questionnaire.

In our study, no statistically significant difference was found in poor SRH for current smokers compared with never smokers. This finding is contrary to previous studies [12, 13, 48], that reported a significant association between current smoking and poor SRH. A possible explanation is that smoking is socially acceptable and is considered a norm in Malaysian culture and society, particularly among men [49]; thus, the relationship between smoking and poor SRH may be confounded. Studies in Greece [35] and Japan [50] have consistently found a strong association between current heavy smokers (>20 cigarettes/day) and poor SRH, while no significant association was found for current light smokers (≤20 cigarettes/day). This may be because heavy smokers are more likely than light smokers to perceive themselves at higher risk of developing smoking-related diseases such as lung cancer or chronic lung disease [51]. Our results showed that former smokers had significantly increased odds of poor SRH compared with never smokers. Former smokers reported higher odds of poor SRH, possibly because of personal health problems that led them to quit smoking [52]. Further study is needed to explore the relations between former smokers and SRH.

In line with previous studies [46, 53, 54] that support the negative effects of alcohol consumption on SRH, our study showed that current drinkers were associated with greater likelihood of poor SRH compared with non-drinkers. However, our results contradict those obtained in Spain [55], which reported better health status among alcohol drinkers than non-drinkers. In Spain, the habit of alcohol consumption has developed within a context of social and leisure activities that contribute to a more positive SRH [56]. The different cultural contexts between Western and Asian countries may account for these differences in SRH. Interestingly, a study in China found a significant association between alcohol drinkers and good SRH but the authors suggested that this finding may have been caused by lack of specific information about the drinking habits of respondents, particularly the number of units consumed and the frequency of alcohol consumption, in the analyses [57]. Results on lifestyle factors vary broadly across studies and populations. In general, individuals who adopt a more proactive lifestyle report better health ratings than those who do not [33, 36, 44, 46, 47].

There is substantial evidence supporting the negative impact of chronic diseases on SRH [3, 16, 34, 58]. People with chronic diseases can experience pain and disability that results in poor SRH. Our study showed that chronic diseases such as asthma, arthritis, hypertension, hypercholesterolemia, and heart disease were strongly associated with poor SRH, as reported in the literature. However, we did not find a significant association between diabetes mellitus and poor SRH. A possible explanation for this is that people with diabetes may also have been diagnosed with hypertension and/or hypercholesterolemia. The presence of these chronic diseases may attenuate the effects of diabetes on SRH, resulting in a finding of non-significance. It is also possible that the respondents in our study who had disbetes mostly seek care from public health clinics that have effective diabetes control programmes and diabetes nurse educator services, resulting in these people having better self-care practices for diabetes management. The findings on diabetes and SRH indicate a need for further investigation.

While this study emphasised the associations between lifestyle, chronic diseases, and SRH, several socio-demographic factors such as age, sex, ethnicity, and residence (urban/rural) were shown to have statistically significant influences on SRH in Malaysia. Older people were more likely to report poor SRH than younger people, which has been shown in other studies [12, 13, 35]. Women were more likely to report poor SRH than men. This finding is related to women being more likely than men to report in their general health assessment a wide range of health-related problems from simple illness or chronic diseases as well as non-health-related problems such as anxiety, depression, or a recent negative life event [59, 60]. Ethnic differences in SRH may be due to cultural differences in how individuals perceive their health status. Other Bumiputeras reported the highest prevalence of poor SRH, compared with other ethnic groups, probably because the majority live in rural areas. People in rural areas were more likely than those in urban areas to rate their health status as poor; this finding may be related to limited accessibility to health care services and lack of financial means to pay for services.

The major strengths of this study are the representativeness and large sample size. The results can be extrapolated accurately and generalised to the Malaysian adult population. Furthermore, the participants were interviewed face-to-face by trained interviewers and the response rate was higher than reported for other studies. However, the study has several limitations that should be noted. The main limitation is the cross-sectional design of the study, which does not allow for determination of causal effect relationships. Another limitation is the possibility of recall bias associated with self-reported data on smoking, alcohol consumption, physical activity, and consumption of fruits and vegetables. Third, the “social desirability” of certain answer categories is a concern in face-to-face interviews. To project a favourable image, respondents may give answers that are deemed more “socially accepted” than their “true” answers. Finally, several other factors found to be associated with SRH in previous studies (e.g., psychosocial and environmental factors) were not investigated in this study.

## Conclusions

The prevalence of poor SRH among Malaysian adults is 20.1 %, which increases with age. Several negative lifestyle behaviours and chronic diseases were significantly associated with poor SRH among Malaysian adults. Additional public health strategies are needed to promote healthy lifestyles and disease prevention interventions should be implemented to improve personal and community health. More preventive measures and health care services targeting chronic diseases are urgently needed, particularly in rural communities. Further research that takes into account the influences of other potential confounders such as psychososial and environmental factors is needed to fully understand the correlates and determinants of SRH.

## Additional file

Additional file 1: Table S1. Percent distribution of self-rated health status (five SRH cetegories), Malaysian adults aged 18 years and older, NHMS 2011 (DOCX 15 kb)
