# Supplementary material for: Lifestyle, chronic diseases and self-rated health among Malaysian adults: results from the 2011 National Health and Morbidity Survey (NHMS)
Source: BMC Public Health. 2015 Aug 6;15:754. doi: 10.1186/s12889-015-2080-z (PMC4527234; doi:10.1186/s12889-015-2080-z)
Supplement: Additional file 1: Table S1. — Percent distribution of self-rated health status (five SRH cetegories), Malaysian adults aged 18 years and older, NHMS 2011 (DOCX 15 kb) [file 12889_2015_2080_MOESM1_ESM.docx]

**Appendix Table 1: Percent distribution of self-rated health status (five SRH cetegories), Malaysian adults aged 18 years and older, NHMS 2011**

| **Self-rated health status** | **Men (n=8588)** | **Women (n=9596)** | **Total (n=18184)** |
| --- | --- | --- | --- |
|  | **n (%)** | **n (%)** | **n (%)** |
|  |  |  |  |
| Very good | 1727 (20.5) | 1741 (18.5) | 3468 (19.4) |
| Good | 5296 (61.1) | 5735 (59.8) | 11031 (60.4) |
| Moderate | 1410 (16.6) | 1939 (19.7) | 3349 (18.2) |
| Not good | 142 (1.6) | 174 (1.9) | 316 (1.8) |
| Very bad | 13 (0.2) | 7 (0.1) | 20 (0.1) |
|  |  |  |  |
